# Editorial: Rare and misdiagnosed neurosurgical conditions volume II

**DOI:** 10.3389/fsurg.2025.1693944

**Published:** 2025-12-11

**Authors:** Moksada Regmi, Chenlong Yang

**Affiliations:** 1 State Key Laboratory of Vascular Homeostasis and Remodeling, Department of Neurosurgery, Peking University Third Hospital, Peking University, Beijing, China; 2 Center for Precision Neurosurgery and Oncology of Peking University Health Science Center, Peking University, Beijing, China; 3 Peking University Health Science Center, Beijing, China; 4 Center for Oculocranial Pressure Instability Disorders, Henan Academy of Innovations in Medical Science (AIMS), Zhengzhou, China

**Keywords:** diagnostic challenges, intracranial neoplasms, hydrocephalus, endoscopic neurosurgery, rare neurosurgical diseases

Rare and misdiagnosed neurosurgical disorders are easy to sideline because each seems singular. Yet as precision neurosurgery matures, the common failure mode across these conditions is surprisingly uniform: delayed recognition, ad-hoc decision-making, and thin follow-up. The contributions in this Research Topic highlight both the promise and the gaps. To change outcomes, we need consistent diagnostic playbooks, disciplined selection of operative corridors, and adjuvant strategies that are prospectively evaluated rather than improvised.

Diagnostic certainty is the first bottleneck. Metagenomic next-generation sequencing (mNGS) has moved from proof-of-concept to routine consultation in several centers for suspected central nervous system infection. In prospective experiences, unbiased mNGS of cerebrospinal fluid has clarified etiologies that eluded standard panels, changed management, and—crucially—done so on a clinically actionable timetable. Results across large cohorts still vary with organism load and bioinformatic thresholds, but the signal is consistent: mNGS is not a last resort; it is an early adjunct when initial microbiology and imaging disagree with the clinical trajectory. Building it into the first 72 h of evaluation for undifferentiated meningoencephalitis—rather than after multiple negative rounds—should be standard (1, 2).

The same logic applies to amebic disease. Balamuthia mandrillaris remains catastrophic, with mortality approaching 90% despite multi-drug regimens. Recommendations emphasize rapid antimicrobial coverage and public-health consultation; yet most patients also die from secondary physiology we can influence: intracranial hypertension, obstructive hydrocephalus, and ventriculitis (3). The case in this Research Topic that used ventriculoscopy with targeted intraventricular lavage highlights a practical point: early cerebrospinal fluid diversion and endoscopic clearance can stabilize the substrate on which antimicrobials must work (Wang et al.). This is not a cure, and the evidence is at the case-series level; still, it argues for a protocolized pathway that couples expedited mNGS, immediate antimicrobial coverage guided by expert consultation, and early surgical management when CSF dynamics deteriorate. A prospective registry dedicated to free-living ameba infections—tracking time-to-diagnosis, neurosurgical interventions, and functional outcomes—would supply the denominator we lack.

Hydrocephalus with an isolated or “trapped” fourth ventricle is another archetype where standardization is overdue. Options span fourth-ventricle shunting, endoscopic third ventriculostomy (ETV), trans- or retro-aqueductal stenting, and tailored shunt configurations—each with different failure modes (4). Contemporary evidence suggests that endoscopic strategies are reasonable first approaches for fourth-ventricle outlet obstruction when the anatomy permits, with shunt or stent rescue for recurrence or complex loculations. Phase-contrast cine MRI adds noninvasive physiology that can shift choices among ETV, stent, and shunt (5). Reporting should use standardized endpoints—time to re-intervention, brainstem compression reversal, and patient-reported function—so the field can compare like with like.

The cavernous sinus illustrates a different challenge: histologic outliers masquerading as common meningiomas. The reclassification of hemangiopericytoma under the solitary fibrous tumor/hemangiopericytoma (SFT/HPC) spectrum—with pathognomonic STAT6 nuclear expression driven by NAB2–STAT6 fusion—has transformed diagnosis and follow-up (6). For lesions that encroach on the cavernous sinus, an endoscopic transpterygoid route can provide direct access while preserving lateral neurovascular structures, but the biology, not the corridor, governs recurrence risk. Evidence from pooled series indicates that adjuvant radiation—often stereotactic radiosurgery—improves local control after subtotal resection, yet does not eliminate the propensity for late intracranial or extracranial metastasis (7). Lifelong surveillance is appropriate. Molecularly informed radiotherapy dosing schemas and prospective registries specific to intracranial SFT/HPC should be the next step, allowing treatment to be tiered by margin status and mitotic index rather than by anecdote (8).

Occasionally, the “rare” diagnosis shifts the operative goal from maximal removal to planned subtotal resection plus focused radiation. The two intracavernous SFT/HPCs in this collection, approached endonasally (with one combined approach), highlight that reality and also the cost of overconfidence: bleeding risk is high and neurovascular adherence can be unforgiving. The literature supports endonasal transpterygoid exposure of the parasellar carotid with satisfactory visualization, but there remains no randomized comparison of corridors, and outcomes hinge on case selection and adjuvant therapy (9, 10). Until stronger comparative data exist, centers should pre-register their intended approach, margin goals, and adjuvant plan and report outcomes transparently; this is the path to learning-health-system evidence for low-incidence skull-base disease.

Not all “rare” problems are deep. Peripheral manifestations can be just as elusive and just as fixable. Median-nerve entrapment from intratendinous gouty tophi is uncommon and easily mislabeled as idiopathic carpal tunnel syndrome when a mass is not evident. Dynamic ultrasound—rather than static imaging alone—can show tendon–nerve conflict in real time and direct both decompression and tophus-targeted therapy (Qu et al.). The point is general: in low-prevalence entities, we should prioritize modalities that demonstrate mechanism (flow, motion, physiological compromise) over those that only catalog morphology. That mindset is as relevant for the aqueduct as it is for the carpal tunnel.

Temporal bone intraosseous arteriovenous malformations remind us how easily vascular anomalies can be overlooked when they sit in bone (11). Here, angiographic planning is not just confirmatory; it is preventive for surgical bleeding and a determinant of corridor. Case-based literature remains sparse, but the pattern is consistent: individualized combinations of resection and embolization tailored to location and feeders, with seizure control or hemorrhage prevention as the relevant outcome (Sharifi et al.).

What, then, should change? First, adopt “diagnostic escalation” early when common tests do not match the clinical course. For suspected CNS infection with negative initial studies, add mNGS within the first hospital days and plan around specimen requirements; for suspected fourth-ventricle isolation, supplement structural MRI with cine flow when it will alter the choice among ETV, stent, and shunt. Second, embed histomolecular typing into skull-base workflows so that SFT/HPCs are distinguished up front and adjuvant radiation is planned prospectively rather than reactively. Third, design follow-up that matches biology: long-horizon imaging for SFT/HPC; early reassessment of CSF dynamics after endoscopic reconstruction of complex hydrocephalus; structured neurological evaluation after infectious ventriculitis with surgical lavage or diversion.

Fourth, measure what matters to patients. For complex hydrocephalus, durability—time to re-intervention or shunt failure—is the relevant endpoint. For intracranial mesenchymal tumors, local control and late metastatic events, not just perioperative morbidity, should drive quality metrics. For rare CNS infections, time from presentation to pathogen-directed therapy and to definitive CSF control are the actionable intervals worth benchmarking.

Finally, we should build the infrastructure that rare conditions demand. A federated, prospective registry for rare neurosurgical entities—pre-specified data elements, standardized imaging uploads, central pathology review where feasible, and planned analyses—would convert isolated case reports into cumulative knowledge. The model is not hypothetical; it exists in adjacent domains. The UK Biobank shows what scale, consent, and governance can look like for secure, cloud-based access to deeply phenotyped, genotyped cohorts (12). Complementary efforts—such as the NeuroVisionaries' NeuroBANK (Somadisk) project that treats clinical data as the substrate for reproducible, privacy-preserving discovery—could be aligned with an outcomes-oriented neurosurgical registry so that sites contribute prospectively and benefit immediately from shared analytics and decision support. Such a platform could also support adaptive studies that test practical questions: in trapped-fourth-ventricle hydrocephalus, does a flow-guided algorithm improve re-intervention-free survival compared with surgeon preference? In SFT/HPC, do margin-driven stereotactic radiosurgery dose bands alter local control without increasing cranial neuropathy?

Open questions should now be trial-shaping and measurable. Can we prospectively validate a rapid diagnostic bundle for undifferentiated meningoencephalitis—integrating unbiased pathogen detection with host-response assays—that shortens time to targeted therapy and lowers disability at discharge? For free-living amebic disease, does a standardized neurosurgical adjunct strategy—early diversion with protocolized endoscopic management of ventriculitis—improve survival when layered onto best available antimicrobials?

In complex hydrocephalus, will an algorithmic, physiology-guided approach that incorporates cine flow and predefined anatomic criteria outperform usual care in re-intervention-free survival and brainstem decompression? For intracranial SFT/HPC, does a risk-adapted adjuvant radiation strategy based on margin status and mitotic activity reduce first-site failure, and should surveillance intervals be individualized by molecular risk rather than fixed calendars?

The studies in this Topic show that many “rare” problems are, in effect, common problems with thinner evidence. The corrective is not more heroics but more consistency: earlier unbiased diagnostics, anatomy-and-physiology-aligned operations, molecularly informed adjuvant care, and outcomes that we can compare across centers. Standardizing the exceptions is how we will shrink the distance between what we know and what we do—one rare case at a time.
